# Nrf2/HO-1–dependent inhibition of ferroptosis underlies the antioxidant effects of 5-O-methylvisammioside in colitis

**DOI:** 10.3389/fimmu.2025.1738408

**Published:** 2026-01-20

**Authors:** Donglin Li, Fagang Xu, Wei Wang, Chuan Jiang, Wenwen Cui, Zhong-an Guan, Chao Gu

**Affiliations:** 1 The First College of Clinical Medicine, Shandong Traditional Chinese Medicine University, Jinan, Shandong, China; 2 Department of Proctology, Dongying People’s Hospital, Dongying Hospital of Shandong Provincial Hospital Group, Dongying, Shandong, China; 3 Department of Gastroenterology, Affiliated Hospital of Shandong University of Traditional Chinese Medicine, Jinan, Shandong, China; 4 Department of Proctology, Affiliated Hospital of Shandong University of Traditional Chinese Medicine, Jinan, Shandong, China

**Keywords:** 5-O-methylvisammioside, ferroptosis, intestinal barrier function, Nrf2/HO-1 pathway, ulcerative colitis

## Abstract

**Background:**

Ferroptosis contributes to epithelial injury and chronic inflammation in ulcerative colitis, yet pharmacologic strategies that durably attenuate ferroptosis and restore barrier function remain limited. 5-O-Methylvisammioside (MeV) is a natural compound with putative antioxidant properties, but its anti-colitic mechanisms are unclear.

**Purpose:**

To determine whether MeV alleviates colitis by activating the Nrf2/HO-1 axis and constraining ferroptosis.

**Methods:**

Acute colitis was induced in male Sprague–Dawley rats using dextran sulfate sodium and animals were randomized to vehicle, DSS alone, two MeV doses, or mesalazine as a benchmark control. Clinical activity, colon length, and histopathology were quantified alongside epithelial barrier readouts (ZO-1, occludin), oxidative stress and ferroptosis markers (lipid reactive oxygen species, malondialdehyde/4-hydroxynonenal, iron accumulation, GPX4, ACSL4), and Nrf2/HO-1 activation, including nuclear translocation of Nrf2. In parallel, erastin-challenged Caco-2 cells were used to test whether MeV directly restrains ferroptosis; Nrf2 knockdown probed pathway dependency.

**Results:**

MeV dose-dependently reduced disease activity and histological damage and partially normalized colon length. Barrier proteins were preserved, and lipid peroxidation and iron overload were diminished. MeV increased Nrf2 nuclear translocation and upregulated HO-1, restored GPX4, and lowered ACSL4, consistent with ferroptosis restraint. *In vitro*, MeV limited erastin-induced lipid ROS and cell injury; Nrf2 silencing blunted these protective effects, supporting pathway involvement. Mesalazine produced improvements of similar magnitude on selected endpoints.

**Conclusion:**

MeV alleviates experimental colitis, at least in part by activating Nrf2/HO-1 to restrain ferroptosis and preserve epithelial barrier integrity. These findings nominate MeV as a ferroptosis-targeting candidate for colitis.

## 1 Introduction

Ulcerative colitis (UC), a refractory and relapsing non-specific inflammatory bowel disease classified by the World Health Organization, exhibits continuous and diffuse involvement typically restricted to the mucosal and submucosal layers. Clinical signs encompass diarrhea, bloody mucopurulent feces, tenesmus, stomach discomfort, and distension (1). Its precise pathogenesis remains elusive but may involve dysregulated immune responses, genetic susceptibility, and gut dysbiosis (2) (3).Globally, UC prevalence exceeds 5 million cases and continues to rise, with patients carrying a substantially elevated colorectal cancer risk compared to healthy populations (4, 5).Current mainstay therapies such as 5-aminosalicylic acid, corticosteroids, and immunosuppressants are severely limited in long-term clinical utility due to drawbacks including pronounced drug resistance, low remission rates, and significant adverse effects.

Numerous studies have shown that herbal medicines contain diverse natural constituents with significant anti-inflammatory and antioxidant properties, indicating broad clinical application prospects (6, 7). The Wuzang Tong Tiao Decoction was optimized from classic traditional Chinese medicinal formulas. It has been used to treat ulcerative colitis for many years and clinical researches have consistently demonstrated its efficacy (8–11). In our previous research, we conducted an in-depth characterization of the chemical constituents, blood-absorbed components, and *in vivo* metabolites of the Wuzang Tong Tiao Decoction using UPLC-QE-HF-MS/MS technology. The identification results revealed that 5-O-methylvisammioside (MeV) ranked among the top compounds in terms of abundance, both in the chemical composition and blood-entry component profiles, suggesting that it is likely one of the primary active ingredients responsible for the formula’s efficacy (12). Previous studies also indicated that one of the therapeutic mechanisms of the Wuzang Tong Tiao Formula in ulcerative colitis involves modulating the Nrf2/HO-1 pathway, regulating oxidative stress and ferroptosis, as well as reshaping the gut microbiota, thereby alleviating intestinal inflammation. 5-O-methylvisammioside (MeV), or 4′-O-β-D-glucosyl-5-O-methylvisamminol, is a chromone extracted from the roots of Saposhnikovia divaricata, with remarkable anti-inflammatory activities (13). Studies have shown that MeV exerts anti-inflammatory effects through pathways such as TNF/MAPK/NF-κB, RAGE/MEK/ERK, and NF-κB/TLR4/COX (14–16). However, these studies did not focus on intestinal inflammation. Only a few reports have indicated that MeV can restore oxidative stress indicators and inflammatory cytokines to normal levels in DSS-induced ulcerative colitis mice (17). Thus, the therapeutic efficacy and mechanism of MeV in ulcerative colitis remain largely unexplored, and no studies to date have investigated its role in ferroptosis. Nevertheless, upon careful review of related literature, we identified several suggestive findings: Research by Xiangyu Li et al. revealed that a combination of compounds isolated from the Schizonepeta and Saposhnikovia herb pair effectively regulated the STAT3/p53/SLC7A11 axis to inhibit ferroptosis. Notably, MeV exhibited the highest peak area in this combination (18). The research conducted by Linxuan Xiao et al. further revealed that the proprietary Chinese medicine Gan-Wei-Kang Tablets protected hepatocytes from H_2_O_2_-induced acute oxidative damage by activating the Nrf2/HO-1 and MAPKs pathways. HPLC quantitative analysis identified MeV as one of the active components in these tablets (19). Moreover, MeV contains an aglycone structure with an α,β-unsaturated ketone moiety, which is known to possess high potential for activating Nrf2.

Based on these findings, we reasonably hypothesize that MeV may be intricately linked to the Nrf2/HO-1 signaling pathway and ferroptosis. The present research seeks investigate the curative effects of MeV on DSS-induced ulcerative colitis in animal and pertinent cellular models, and to further explore whether its underlying mechanism involves ferroptosis mediated by the Nrf2/HO-1 pathway. The outcomes of this research are expected to provide valuable scientific insights for the management of ulcerative colitis and inflammation-associated premalignant colorectal lesions.

## 2 Materials and methods

### 2.1 Materials and reagents

MeV were acquired from Shanghai Yuanye Bio-Technology Co., Ltd (>98% purity by HPLC, Shanghai,China). Dextran sulfate sodium DSS (MW: 36–50 kDa) was purchased from MP Biomedicals (California, USA). Sustained-release mesalazine granules (Etiasa) were obtained from Shanghai Aipu Pharmaceutical Co. Ltd. (Shanghai, China). The qualitative detection kit for fecal occult blood (pyramidon method) was acquired from Solarbio (Beijing, China). ELISA kits for inflammatory factors were provided by Jianglai Biotechnology Co. Ltd. (Shanghai, China). Assay kits for MDA, GSH, 4-HNE, and Fe were obtained from Nanjing Jiancheng Bioengineering Institute (Nanjing, China). Antibodies against occludin and ZO-1 were purchased from Wuhan Proteintech Biotechnology Co. Ltd. (Wuhan, China). Primary antibodies against the other markers were obtained from Abcam (Cambridge, UK). Primers for the aforementioned targets were synthesized by Tsingke Biotechnology Co. Ltd. (Beijing, China). Reverse transcription and PCR kits were supplied by Albatross Biotechnology Co. Ltd. (Guangzhou, China). Human colorectal adenocarcinoma Caco-2 cells were procured from Wuhan Boster Biological Technology Co. Ltd. (Wuhan, China). Trypsin was obtained from Thermo Fisher Scientific (Waltham, MA, USA). The CCK-8 assay kit was provided by Albatross Biotechnology Co. Ltd. (Guangzhou, China). The Erastin and Fer-1 were acquired from MedChemExpress (Shanghai, China). Lipofectamine 2000 transfection reagent was obtained from Invitrogen (California, USA). Small interfering RNA (siRNA) was synthesized by Huzhou Hippo Biotechnology Co. Ltd. (Huzhou, China).

### 2.2 Experimental animals

Specific pathogen-free (SPF) male Sprague-Dawley rats (n=60), weighing 200 ± 20 g (6 weeks old), were obtained from Beijing Vital River Laboratory Animal Technology Co., Ltd. (Production License No. SCXK (Jing) 2021-0006. Animals were housed at the Animal Experiment Center of Shandong University of Traditional Chinese Medicine Affiliated Hospital under regulated circumstances (temperature: 20-26°C; relative humidity: 40-70%) with a 12-hour light/dark cycle.

### 2.3 UC model induction and drug intervention

After acclimatization, rats were randomized into six groups (n=10): (1) normal control, (2) DSS-induced model control, (3) mesalazine, (4) low dose MeV-treated group,and(5) high dose MeV-treated group. UC was induced with 3% DSS (10 days). Based on previously published literature related to MeV and preliminary exploratory experiments, the administered doses for the low-dose and high-dose MeV groups in rats were determined as 3.5 mg/kg/d and 7 mg/kg/d, respectively (20, 21). The dosage of mesalazine was determined to be 0.36 g/kg/day, calculated based on the clinical human dosage and the human-to-rat conversion factor.For the first 3 days of the experiment, all groups of rats received standard food and water. The low- and high-dose MeV treatment groups were administered their respective doses via intraperitoneal injection once daily, while the mesalazine group received its dose via oral gavage once daily. Starting from day 4, except for the control group, the drinking water for all other groups was replaced with a 3% DSS solution (prepared by dissolving 3 g of DSS in 100 mL of double-distilled water). Concurrently, the corresponding drug interventions (MeV or mesalazine) were continued in their respective treatment groups until the end of day 14.

### 2.4 DAI score

During the experimental period, daily measurements of food intake, body mass, stool consistency, and fecal occult blood were performed to compute the disease activity index (DAI) according to the scoring criteria presented in **Table 1**.

**Table 1:** DAI scoring criteria.

Weight loss rate(%)	Blood in the stool	Stool properties	Scoring value (points)
<1	Negativity-	Normal	0
1-5	Occult Blood+	Fluffy and moldable	1
5-10	Occult blood++	Loose stool with scanty mucus	2
10-15	Occult blood+++	Meager stool	3
>15	Bloody stools in the flesh	Watery stool	4

DAI score = (body mass loss score + stool trait score + blood in stool score)/3.

### 2.5 Colonic histopathological assessment and organ index calculation

After 24-hour fasting, the rats were anesthetized (Zoletil-50/Xylazine, 80/20 mg/kg, i.p.). Blood was extracted from the abdominal aorta, centrifuged, and the serum was preserved at -80 °C. The entire colon was excised, measured, photographed, and scored for mucosal damage (CDMI criteria). The detailed CDMI scoring criteria are presented in **Table 2**. Tissues were fixed in 4% PFA for H&E staining (4-μm sections). The spleen and thymus were weighed to determine the organ indices (mg/g).

**Table 2:** CDMI scoring criteria.

Damage to the intestinal mucosa	Score value(points)
No damage	0
Mild congestion and edema, smooth surface, no ulceration	1
Ulceration present without congestion or wall thickening	2
Ulceration with localized inflammation	3
≥2 ulcerated areas with inflammation	4
Major lesions extending >1 cm along colon length	5
Injury extension >2cm	Increase 1 point for every 1cm

### 2.6 AB-PAS staining

Colon slices fixed in paraffin underwent AB-PAS staining to evaluate the quantity of goblet cells. After deparaffinization and rehydration, sections were stained with Alcian blue, oxidized with periodic acid, and treated with Schiff’s reagent. Following dehydration and mounting, acidic mucins appeared blue and neutral mucin/glycogen red under the microscope.

### 2.7 *In Vitro* cell culture

Caco-2 cells were resuspended and maintained under standard conditions (37 °C, 5% CO_2_, 95% air, and saturated humidity) in DMEM basal medium supplemented with 20% fetal bovine serum and 1% penicillin-streptomycin. Cells were passaged at 80-90% confluence using a 1:2 split ratio for the initial post-thawing passage and 1:3 thereafter.

### 2.8 CCK-8 analysis

Caco-2 cells were plated in 96-well plates and divided into nine groups: control group, groups treated with different concentrations of MeV (5, 10, 20, 40,60,80,100 and 200uM). After the cells had fully adhered, cell viability was measured at 24 h via microplate absorbance, and OD values were used to calculate survival rates and determine nontoxic concentrations.

The cells were allocated to a control group, Erastin group, and groups treated with different non-toxic concentrations of MeV (5,10,20,40uM) + Erastin, after the cells were completely attached to the wall, except for the control group. The remaining groups were treated with Erastin and their respective concentrations of MeV. The cell viability of each group was detected after 24h, and the effective concentration of MeV were screened.

### 2.9 Establishment of a Caco-2 cell monolayer model

Caco-2 cells were seeded at a density of 7×10^4^ cells/cm³ on collagen-coated polytetrafluoroethylene Transwell plates (24-well format, pore size 0.4 μm, membrane area 0.33 cm²), with 600 μL of complete culture medium added to the basal compartment (BL) and 100 μL of cell suspension inoculated into the apical compartment (AP). The medium was replaced within 16 hours post-seeding to prevent multilayer formation due to cell overgrowth. During the first week, the culture medium was changed every other day, and from the second week onward, it was replaced daily until day 21. Starting on day 3 post-seeding, transepithelial electrical resistance (TEER) was measured every 3 days using a voltohmmeter, calculated according to the formula TEER = (R − R_0_) × S, where TEER represents the transepithelial electrical resistance (Ω·cm²), S is the effective membrane area of the insert (cm²), R is the mean resistance value (Ω) measured at three fixed positions of the cell-coated insert, and R_0_ is the mean resistance value (Ω) of a blank insert without cells measured at three fixed positions. Simultaneously, from day 3 onward, alkaline phosphatase (ALP) activity in both AP and BL compartments was assessed every 3 days to evaluate the integrity and functionality of the intestinal epithelial monolayer model.

### 2.10 Cell grouping and drug intervention

Caco-2 cells were seeded in 6-well plates and divided into five groups: CON, Erastin, the low-dose and high-dose MeV + Erastin treated groups, and ferroptosis inhibitor (Fer-1 10μmol/L) group. Measurements of various parameters were initiated after 24 hours of treatment.

### 2.11 ELISA for inflammatory factors and measurement of oxidative stress level

ELISA for the detection of IL-6, IL-8 and TNF-a: Rat serum and cell samples were taken, and the expression levels of inflammatory mediators were detected in accordance with the procedure described in the ELISA kit; The expression levels of MDA, GSH, SOD, CAT and Fe2+ were measured in both colon tissue and cell samples. The specific procedures were performed in accordance with the manufacturer’s guidelines for each reagent kit.

### 2.12 The mitochondria was observed by transmission electron microscope

Colon tissue or cells were removed, and 2.5% glutaraldehyde was fixed for 6h, then poured into PBS buffer and 1% acetic acid fixed for 2h. Dehydrated and immersed in the relevant reagents, embedded in an oven at 40°C for 12h, and in an oven at 60°C for 48h. The embedded blocks were cut into ultrathin sections (70 nm thick). Morphological changes in mitochondria stained with lead and uranium were observed using electron microscopy, and the images were recorded using a digital camera.

### 2.13 Immunohistochemistry

The colon tissues were fixed, sectioned, and processed for immunohistochemistry (antigen retrieval, blocking, primary antibody at 4°C overnight, secondary antibody, and DAB/fluorescence detection). After counterstaining and mounting, protein localization was analyzed microscopically and the positive cell rates were quantified using ImageJ software.

### 2.14 Western blotting analysis

Total protein was extracted (RIPA lysate + protease inhibitor) and quantified. After denaturation (Loading Buffer and boiling), the samples were separated by SDS-PAGE (based on the target MW) and transferred to PVDF membranes. The membranes were blocked (5% skim milk for 1hour at RT), incubated with primary antibodies (4°C overnight), and then with biotinylated secondary antibodies (1h, RT). Protein bands were seen by enhanced chemiluminescence and analyzed with ImageJ software.

### 2.15 RT-qPCR

For RT-qPCR, 10 mg of fresh colon tissue was homogenized in 500 μL of lysis buffer (high-speed, low-temperature), centrifuged (12,000 rpm, 5 min), and the supernatant was collected. Alternatively, cells were lysed by repeated pipetting. Total RNA was extracted using a kit, reverse-transcribed into cDNA, and amplified by qPCR using target-specific primers. β-actin served as an internal control. The primer sequences for rat colon tissue and Caco-2 cells are listed in **Tables 3**, **4**.

**Table 3:** Primer sequence information for rat colon tissue.

Primer name	Primer sequence (5’-3’)	Product length/bp
Rat-claudin-1-F	CTGGGGACAACATCGTGACTG	191
Rat-claudin-1-R	GGTGGACACAAAGATTGCGAT	191
Rat-Occludin-F	ACAGACCCAGACCACTATGAAAC	243
Rat-Occludin-R	CTCTTCGCTCTCCTCTCTGTAGT	243
Rat-GPX4-F	CCGACGTAAACTACACTCAGCTA	118
Rat-GPX4-R	TCTTGATTACTTCCTGGCTCCTG	118
Rat-FTH1-F	GCCCTGAAGAACTTTGCCAAATA	139
Rat-FTH1-R	AGTCATCACGGTCAGGTTTCTTT	139
Rat-SLC7A11-F	CGGGGTTGGCTTCCTTATCA	192
Rat-SLC7A11-R	GGGCAGATGGCCAAGGATTT	192
Rat-Nrf2-F	GATCAGGCTCAGTCACTCGATAG	117
Rat-Nrf2-R	ACACTGTAACTCGGGAATGGAAA	117
Rat-HO-1-F	CAGGGTGACAGAAGAGGCTAAGA	246
Rat-HO-1-R	TGGGATGAACTAGTGCTGATCTG	246
Rat-actin-F	AGATCAAGATCATTGCTCCTCCT	174
Rat-actin-R	ACGCAGCTCAGTAACAGTCC	174

**Table 4:** Primer sequence information of Caco-2 cells.

Primer name	Primer sequence (5’-3’)	Product length/bp
Homo-claudin-1-F	GAAGACGATGAGGTGCAGAAGAT	161
Homo-claudin-1-R	CCAAATTCGTACCTGGCATTGAC	161
Homo-Occludin-F	CCAATGTCGAGGAGTGGGTTAAA	213
Homo-Occludin-R	AGTCATCCACAGGCGAAGTTAAT	213
Homo-GPX4-F	GTAAACTACACTCAGCTCGTCGA	119
Homo-GPX4-R	TTGATCTCTTCGTTACTCCCTGG	119
Homo-FTH1-F	GAGAGGGAACATGCTGAGAAACT	177
Homo-FTH1-R	CAGTTTGTGCAGTTCCAGTAGTG	177
Homo-SLC7A11-F	CTTTCAAGGTGCCACTGTTCATC	106
Homo-SLC7A11-R	ACGAAGCCAATCCCTGTACTAAA	106
Homo-Nrf2-F	AGCAAGTTTGGGAGGAGCTATTA	223
Homo-Nrf2-R	GAGAGGATGCTGCTGAAGGAATC	223
Homo-HO-1-F	TCAGGCAGAGGGTGATAGAAGAG	104
Homo-HO-1-R	GCTCTGGTCCTTGGTGTCATG	104
Homo-β-actin-F	CCTTCCTGGGCATGGAGTC	189
Homo-β-actin-R	TGATCTTCATTGTGCTGGGTG	189

### 2.16 Small interfering RNA (siRNA) was employed to knockdown Nrf2 expression

Three different Nrf2-targeting siRNA sequences were designed and transfected into Caco-2 cells using Lipofectamine 2000. Cells were inoculated at a density of 3×10^5^ cells per well in 2 mL of antibiotic-free media 24 h before transfection. For each transfection complex, 10μL of 20μM siRNA was diluted in 250μL Opti-MEM^®^ medium and mixed gently. Independently, 10μL of Lipofectamine™ 2000 was diluted in 250μL of Opti-MEM^®^ and incubated for 5 minutes. The diluted siRNA was subsequently combined with the Lipofectamine™ 2000 mixture, carefully combined through pipetting and incubated for 20 minutes at ambient temperature to facilitate complex formation. Transfection complexes (500μL/well) were added dropwise to 6-well plates containing the seeded cells. After gentle rocking to ensure even distribution, The cells were incubated for 6 hours at 37 °C in a humidified environment containing 5% CO_2_ before being replaced with fresh complete medium. Transfection efficiency was assessed 48 h post-transfection by fluorescence microscopy. Fluorescence micrographs are included in the Results section. The efficacy of the siRNA was evaluated using Nrf2 mRNA levels (RT-qPCR), with the most effective construct selected for further use. Primer sequences are enumerated in **Table 5**.

**Table 5:** SiRNA sequence.

Primers	Sequence (5’→3’)	Tm (°C)
hNRF2 si-1 sense	GGUUGAGACUACCAUGGUUTT	56.1
hNRF2 si-1 antisense	AACCAUGGUAGUCUCAACCAG	58
hNRF2 si-2 sense	GCCCAUUGAUGUUUCUGAUTT	54.1
hNRF2 si-2 antisense	AUCAGAAACAUCAAUGGGCCC	58
hNRF2 si-3 sense	GCAGUUCAAUGAAGCUCAATT	54.1
hNRF2 si-3 antisense	UUGAGCUUCAUUGAACUGCTC	56.1

Caco-2 cells were cultivated in six-well plates and categorized into 4 groups: Erastin + Si-N C, Erastin + MeV+ Si-NC, Erastin+Si-Nrf2 and Erastin+MeV+Si-Nrf2 groups. After the cell density reached 60%, each small interference group was added to the ready-made transfection complex, and the empty vector group was added to an equal volume of transfection reagent without Nrf2Si-RNA. After 6 h, each group was changed to complete medium, and the indices were detected after 48 h.

### 2.17 Experimental details and statistical methods

In the animal experiments, stratified randomization was employed for group allocation. Briefly, each rat was first individually marked, followed by measurement of body weights. All animals were then ranked from heaviest to lightest to generate a sequential list. The sorted list was subsequently divided into blocks of five consecutive rats, resulting in a total of 10 blocks. An online randomization tool was used to generate a random sequence of numbers 1 to 5, which was then mapped to corresponding group identifiers (e.g., C, A, E, B, D). Rats within each block were assigned to the five groups according to this sequence until all blocks had been processed. This stratified randomization approach ensures balanced baseline characteristics among the experimental groups. The sample size for each group was determined *a priori* using power analysis based on preliminary data from a pilot study (effect size d = 1.2, α = 0.05, power = 0.8) conducted with 4 animals per group, which indicated a minimum requirement of 7 animals per group to detect significant differences in the primary outcome (DAI score). Considering potential mortality during DSS induction and the need for sufficient tissue for multiple assays, we included 10 animals per group at the start of the experiment to ensure robust statistical analysis. This sample size is also consistent with common practice in similar colitis intervention studies. Statistical analysis was performed using GraphPad Prism (version 9.5.1) and intergroup significance was analyzed by ordinary one-way ANOVA. Figures were prepared using Adobe Illustrator 2021.

## 3 Results

### 3.1 MeV effectively improves the pathological status and Inflammatory response of UC rats

**Figure 1:**
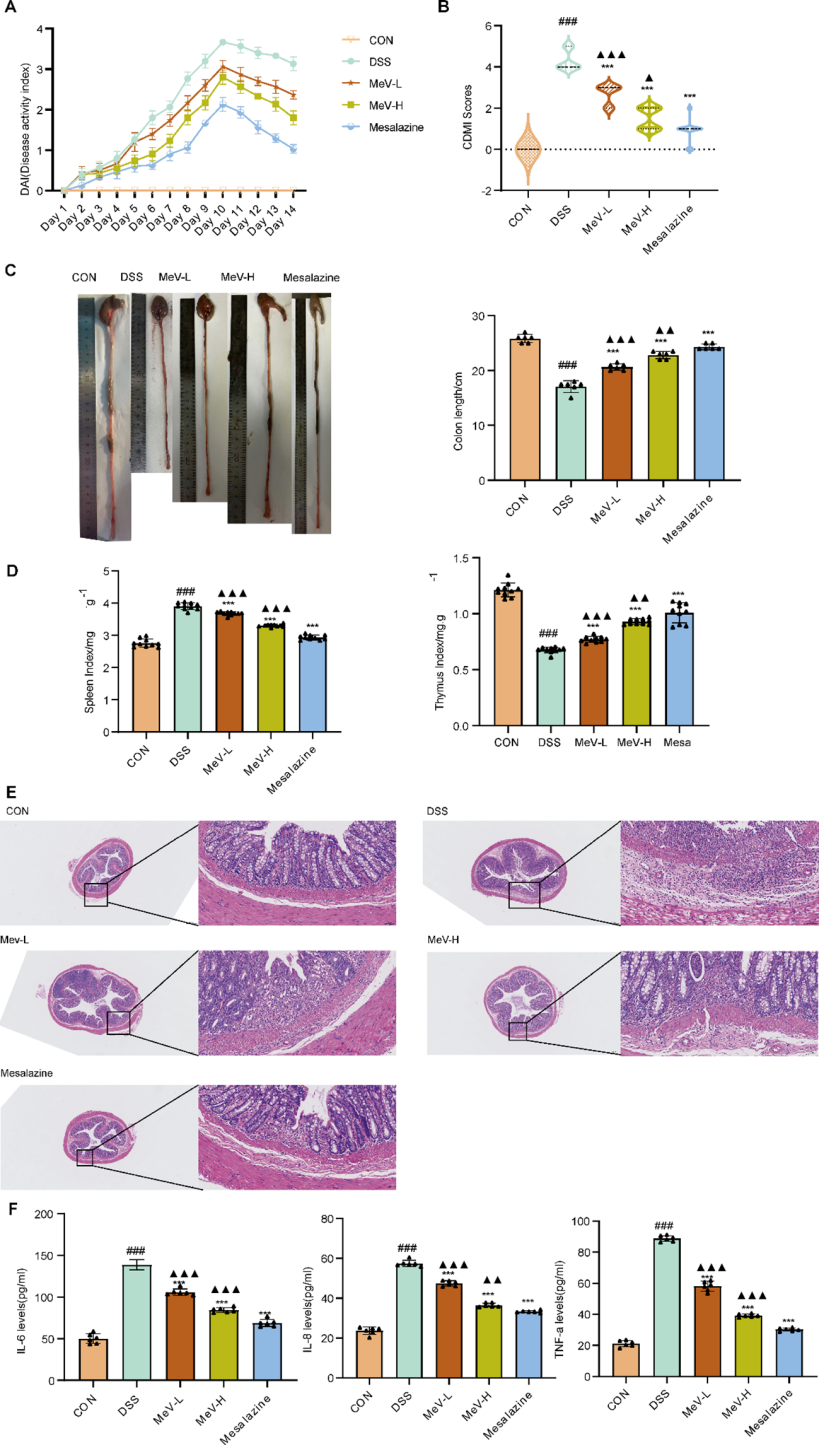
MeV improves the pathological status and inflammatory response of UC rats. **(A)** DAI scores. **(B)** CDMI scores. **(C)** Colon length. **(D)** Organ index (spleen, thymus). **(E)** HE staining images. **(F)** Inflammatory factors in rat colon tissue. Data are presented as mean ± SD. ###p < 0.001 versus Normal group; and ***p < 0.001 versus DSS group.

Control rats showed normal activity, grooming, and weight gain with healthy stools. The model rats developed severe UC symptoms: lethargy, hunched posture, reduced food intake, mucoid/bloody diarrhea, and significant weight loss (peak at day 10). All treatment groups showed a milder weight reduction during DSS exposure and faster post-withdrawal recovery (stool normalization and improved activity), with mesalazine and high-dose MeV exhibiting optimal efficacy. MeV effectively alleviated UC symptoms, as reflected by the DAI scores (**Figure 1A**).

Colon length measurements showed significant differences between the groups (**Figure 1C**). Compared to the controls, the DSS group had markedly shortened colons (p< 0.001). All treatments significantly improved colon length (p < 0.001). Macroscopically, DSS-treated rats exhibited severe adhesions, ulcers, congestion, and thickening (CDMI score ↑, p < 0.001 vs. control). All treatments reduced mucosal damage (CDMI score ↓, p < 0.001), with high-dose MeV and mesalazine showing near-normal mucosa, mild edema, no extensive ulcerations, and no significant intestinal wall thickening (**Figure 1B**).

Relative to the CON group, the DSS rats demonstrated markedly increased spleen indices (p<0.001), along with a markedly reduced thymus index (p<0.001) (**Figure 1D**). Conversely, all treatment groups demonstrated reductions in spleen indices, and an increased thymus index relative to the DSS group. Critically, the mesalazine and high-dose MeV groups achieved statistical significance across all four organ indices compared with the DSS group.

H&E staining demonstrated intact colonic mucosa with normal crypts, glands, and abundant goblet cells in the CON rats, whereas DSS-treated rats exhibited severe mucosal ulceration, crypt/gland atrophy, goblet cell depletion, and dense inflammatory infiltration. Low-dose MeV partially ameliorated these changes, but retained architectural disruption and moderate inflammation, whereas high-dose high showed improved gland/crypt organization and reduced inflammation. The high-dose MeV and the mesalazine group both exhibited significant amelioration in mucosal architecture, including crypts, glands, and goblet cells (**Figure 1E**).

In comparison with the CON group, the levels of IL-6,IL-8 and TNF-α in the DSS rats were significantly increased (p < 0.001), however, compared with the DSS rats, the levels of these in the various medication groups were decreased to different degrees (p < 0.001) (**Figure 1F**).

### 3.2 MeV preserves intestinal mucosal barrier integrity in UC rats

**Figure 2:**
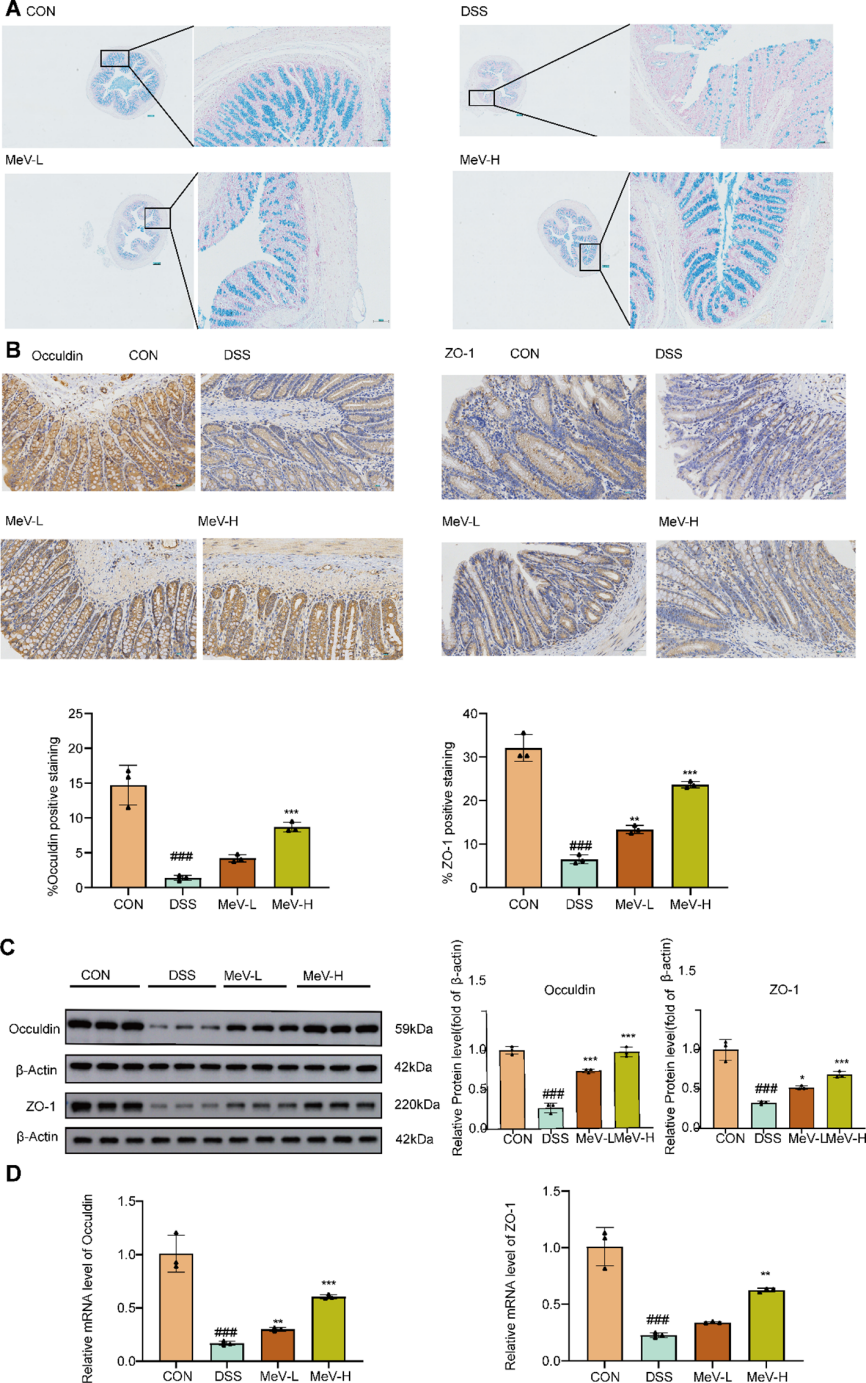
MeV preserves intestinal mucosal barrier integrity in UC rats. **(A)** Alcian blue staining images. **(B)** Immunohistochemistry images and quantification of Occuldin and ZO-1. **(C)** Western blot and quantification of Occuldin and ZO-1. **(D)** Relative mRNA levels of Occuldin and ZO-1. Data are presented as mean ± SD. ###p < 0.001 versus Normal group; *p < 0.05, **p < 0.01, and***p < 0.001 versus DSS group.

Alcian blue staining (**Figure 2A**) showed that the blank control group had normal mucosa with abundant goblet cells, whereas the DSS group displayed severe mucosal damage and goblet cell loss. Both low and high-dose MeV groups demonstrated restoration of mucosal integrity, characterized by significantly increased goblet cell numbers and improved tissue architecture, with the high-dose group exhibiting more pronounced regenerative effects.

Immunohistochemistry showed strong Occludin/ZO-1 expression in the CON group, but a marked reduction in the DSS rats. Treatment with low and high-dose MeV restored the expression in a dose-dependent manner. ImageJ quantification confirmed these trends (**Figure 2B**).

Western blotting: The DSS group showed significantly reduced Occludin and ZO-1 (P<0.001) levels compared to controls. In comparison to the DSS rats, both low and high-dose MeV significantly upregulated the expression of Occludin (P<0.001for both) and ZO-1 (P<0.05, P<0.001) (**Figure 2C**).

RT-qPCR: The DSS rats showed suppressed levels of Occludin and ZO-1 (P<0.001 for both). Both low and high-dose MeV significantly restored Occludin levels (P<0.01,P<0.001), The low-dose MeV showed a slight increase in ZO-1 expression, however, the change was not statistically significant when compared to the DSS rats (P>0.05). In contrast, the high-dose MeV demonstrated a significant upregulation of ZO-1 expression relative to the DSS rats (P<0.01) (**Figure 2D**).

### 3.3 Levels of oxidative stress in animal tissues

**Figure 3:**
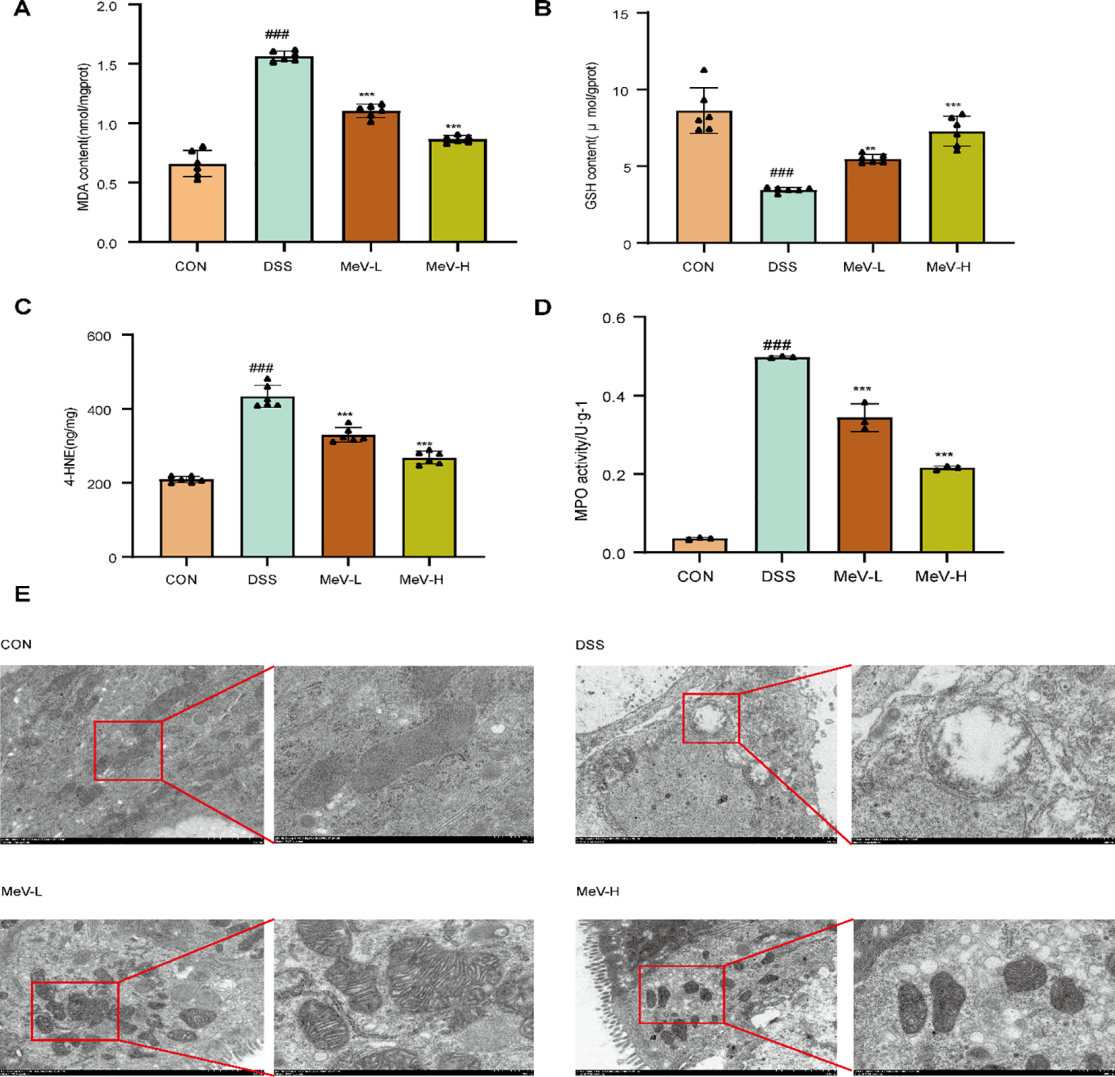
MeV stabilizes oxidative stress levels, MPO activity, and mitochondrial status in rats. **(A)** MDA content. **(B)** GSH content. **(C)** 4-HNE content. **(D)** MPO activity. **(E)** Mitochondrial morphology observed by TEM (×7.0K; × 20.0K). Data are presented as mean ± SD. ###p < 0.001 versus Normal group; **p < 0.01, and ***p < 0.001 versus DSS group.

The DSS treatment markedly elevated the levels of antioxidant indicators (MDA, 4-HNE and Fe; P<0.001) and decreased GSH levels (P<0.001) compared with the controls. All MeV doses reversed these effects, with the MeV-H exhibiting the strongest enhancement (P<0.001) (**Figures 3A-C**). The MPO activity in the DSS group was significantly higher than that in the CON group. Intervention with MeV reduced MPO expression, shifting it closer to the level observed in the CON group (**Figure 3D**).

### 3.4 MeV can effectively inhibit ferroptosis of UC rats

**Figure 4:**
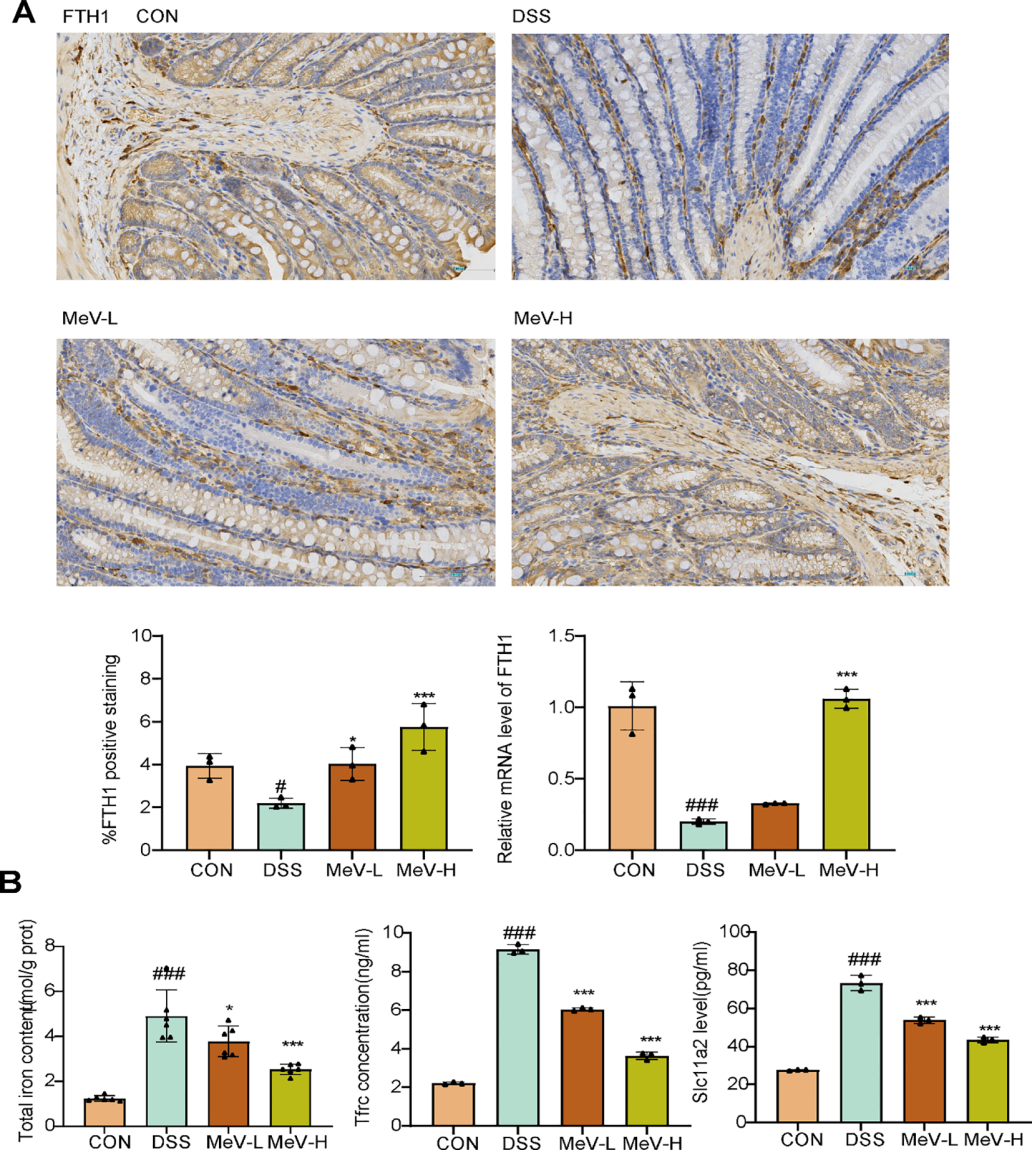
MeV restored iron metabolism homeostasis. **(A)** Immunohistochemistry and RT-PCR results of FTH1. **(B)** Fe,Tfrc and Slc11a2 level. Data are presented as mean ± SD. # p < 0.05,###p < 0.001 versus Normal group; *p < 0.05, and ***p < 0.001 versus DSS group.

The Fe content in the DSS rats was significantly higher than that in the CON group (P < 0.001), whereas the Fe content in the MeV-L and MeV-H groups were significantly lower than that in the DSS rats (P < 0.05, P < 0.001) (**Figure 4B**). Compared with the CON group, the FTH1 level was significantly decreased in the Erastin group, whereas the levels of Tfrc and Slc11a2 were markedly increased. Intervention with low and high-dose MeV reversed the trends observed in the Erastin group to varying degrees, thereby restoring iron metabolic homeostasis (**Figures 4A, B**).

TEM analysis of mitochondrial morphology in rat colonic tissues across groups, with images captured digitally, revealed that mitochondria in the control group were abundant and exhibited normal morphology, characterized by a clearly defined double-membrane structure with distinct outlines, as well as densely packed and regularly arranged cristae; in contrast, DSS group displayed a significant reduction in mitochondrial number accompanied by structural damage, manifested as swollen organelles exhibiting dissolution of the double-membrane structure with loss of clear outlines, along with fragmented, disorganized cristae that were partially to completely lost. Compared to the DSS rats, all treatment groups exhibited increased mitochondrial numbers and reduced swelling. While the double-membraned structure remained indistinct in the low-dose MeV groups, occasional samples in the high-dose MeV group displayed discernible double membranes. These groups also demonstrated an increase in the number of mitochondrial cristae with improved structural definition; however, most cristae exhibited dilation, and mitochondrial morphology showed marked improvement relative to the DSS group (**Figure 3E**).

**Figure 5:**
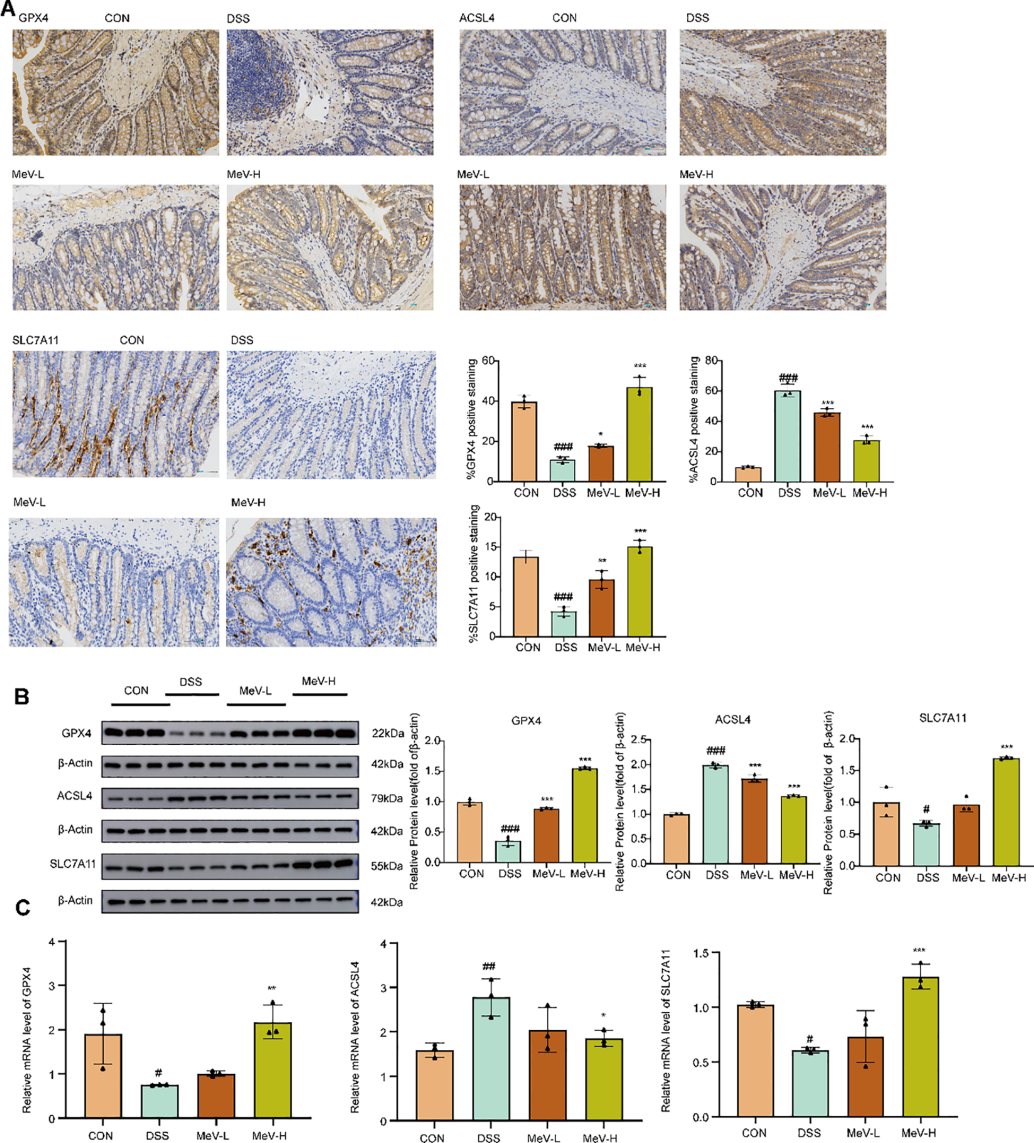
MeV can effectively inhibit ferroptosis in colon tissues of UC rats. **(A)** Immunohistochemistry images and quantification of GPX4, ACSL4 and SLC7A11. **(B)** Western blot and quantification. **(C)** Relative mRNA levels. Data are presented as mean ± SD. #P<0.05, ## p < 0.01, ###p < 0.001 versus Normal group; *p < 0.05, **p < 0.01, and***p < 0.001 versus DSS group.

Immunohistochemistry showed strong GPX4, ACSL4, and SLC7A11 expression (brown staining) in controls, which was markedly reduced in DSS rats. All treatments significantly increased protein expression compared with DSS (**Figure 5A**).

Western blotting revealed that DSS significantly decreased GPX4 (P<0.001), SLC7A11 (P<0.05) and increased ACSL4(P<0.001) compared with the controls. Compared to the DSS group, both MeV-L and MeV-H groups significantly reversed the levels of GPX4 and ACSL4 (P<0.001 for all). Low-dose MeV treatment upregulated SLC7A11 expression, but the difference was not statistically significant compared to the DSS rats (P>0.05), whereas the high-dose MeV group showed highly differences relative to the DSS rats (P<0.001) (**Figure 5B**).

RT-qPCR showed that DSS dramatically reduced GPX4, SLC7A11 and increased ACSL4 mRNA levels (P<0.05, P<0.05, P<0.01). All treatments upregulated GPX4 and SLC7A11 relative to the DSS group whereas only high-dose MeV showed significant differences (P<0.01, P<0.001). Both MeV-L and MeV-H treatments reduced ACSL4 expression, but only the MeV-H group showed statistically significant differences (P<0.05) (**Figure 5C**).

### 3.5 MeV can regulate Nrf2/HO-1 signaling pathway

**Figure 6:**
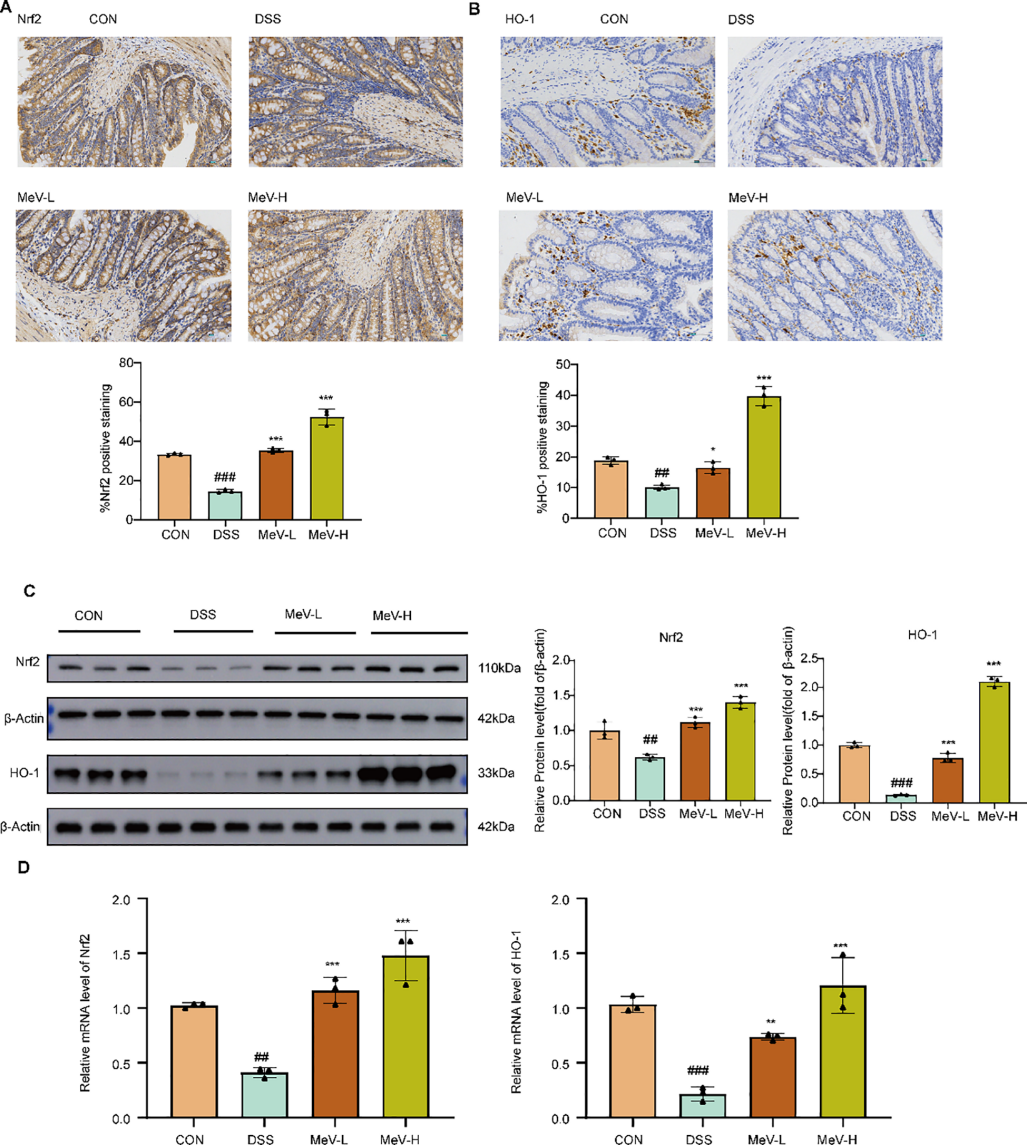
MeV can regulate Nrf2/HO-1 signaling pathway. **(A, B)** Immunohistochemistry images and quantification of Nrf2 and HO-1. **(C)** Western blot and quantification of Nrf2 and HO-1. **(D)** Relative mRNA levels of Nrf2 and HO-1. Data are presented as mean ± SD. #P<0.05, ## p < 0.01, ###p < 0.001 versus Normal group; *p < 0.05, **p < 0.01, and ***p < 0.001 versus DSS group.

Immunohistochemical, WB, and RT-qPCR results consistently indicated that DSS markedly decreased the expression of Nrf2 and HO-1 at both the protein and mRNA levels (All differences were statistically significant vs. the CON group). Both low and high-dose MeV groups markedly restored Nrf2 and HO-1 expression, and demonstrated dose-dependent differences relative to the DSS group. These findings indicate MeV’s dose-dependent regulation of the Nrf2/HO-1 pathway in UC rats (**Figure 6**). HO-1 coordinates an antioxidant program characterized by the restoration of GPX4/SLC7A11, reduction of lipid peroxidation/iron overload, and overall improvement of barrier function.

### 3.6 Safety and effective concentration of MeV in caco-2 cells

The CCK-8 assay findings demonstrated that cell viability values exhibited no significant alterations when compared to the CON group following treatment with MeV at concentrations of 5, 10, 20, and 40 μM, suggesting that MeV within this concentration range exerts no cytotoxic effects. In contrast, under treatment with the four concentrations of 60, 80, 100, and 200 μM MeV, cell viability values exhibited a decrease relative to the CON group, with the differences becoming more pronounced as concentrations increased. This indicates that MeV within this concentration range induces toxic effects on Caco-2 cells and inhibits cell viability.

**Figure 7:**
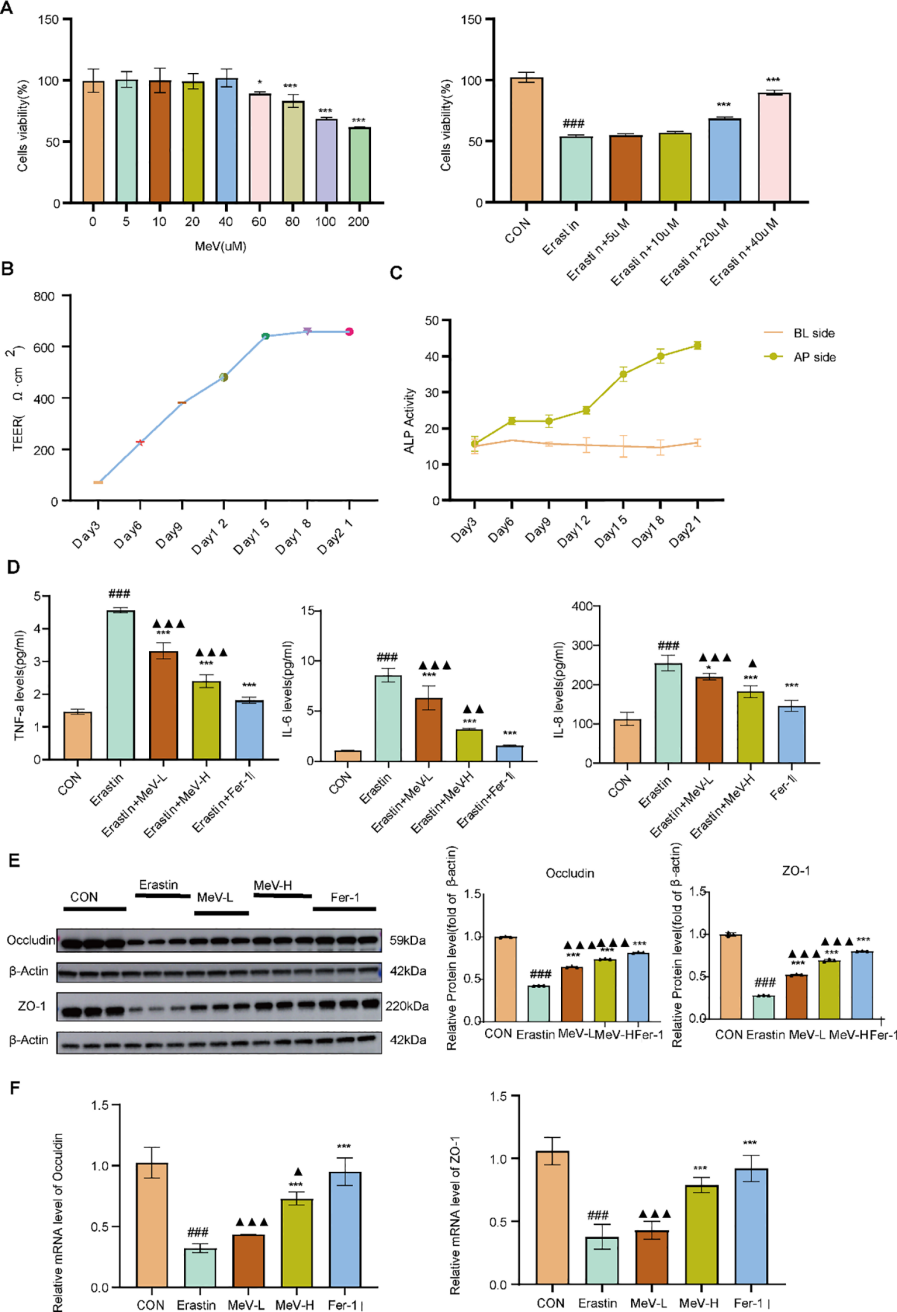
CCK-8 Assay, Establishment of a Caco-2 Cell Monolayer Model;the Effects of MeV on Barrier Function and Inflammatory Response. **(A)**CCK8 analysis. **(B, C)** TEER value and ALP activity of monolayer cell model. **(D)** level of inflammatory factors. **(E, F)** Western blotting and RT-qPCR analyses of occuldin and ZO-1. Data are presented as mean ± SD. #P<0.05, ## p < 0.01, ###p < 0.001 versus CON group; *p < 0.05, and ***p < 0.001 versus Erastin group. ▴P<0.05, ▴▴p < 0.01, ▴▴▴p < 0.001 versus Fer-1 group.

With the exception of the control group, all cell groups were treated with Erastin. Based on the previous CCK-8 results, MeV at the safe concentrations of 5, 10, 20, and 40 μM was selected for administration. After 24 hours of treatment, cell viability values were measured using the CCK-8 assay. The results demonstrated that compared to the CON group, the Erastin-treated group exhibited a significant reduction in cell viability. In contrast, MeV treatment groups at various concentrations showed varying degrees of improvement in cell viability. However, the differences observed in the 5uM and 10uM groups were not statistically significant when in comparison to the Erastin group (P>0.05), whereas at concentrations of 20 and 40 uM, cell viability values were significantly enhanced relative to the Erastin group (P<0.001). Therefore, 20Um and 40uM were selected as the MeV-L and MeV-H groups, respectively, for subsequent experiments (**Figure 7A**).

### 3.7 Establishment and validation of a caco-2 cell monolayer model

As shown in **Figure 7B**, during the initial stage of modeling, the Caco-2 cell monolayer exhibited low TEER values. Throughout the formation process, the TEER gradually increased, with the most significant increase observed during the first 15 days. The rate of increase slowed between days 15 and 21, eventually stabilizing at 658 Ω·cm². This indicates that the Caco-2 cell monolayer had developed excellent integrity. During the modeling process, Caco-2 cells differentiate to form AP and BL sides. ALP is a marker enzyme localized at the AP side of intestinal epithelial cells. As the Caco-2 monolayer forms, ALP continuously accumulates on the AP side, creating a significant gradient compared to the BL side, thereby conferring polarized characteristics to the cellular model. Measuring ALP activity at various stages reflects the biochemical properties and cellular polarity of the Caco-2 model. As seen in **Figure 7C**, with the differentiation of the Caco-2 monolayer, ALP activity on the AP side gradually increased, while it remained largely unchanged on the BL side. By the late stage of differentiation (days 15–21), a significant difference in ALP activity between the two sides was observed, indicating the establishment of distinct polarization in the Caco-2 cell model. At this point, both integrity and polarity met the required standards, demonstrating that the Caco-2 cells had formed a complete and tightly integrated monolayer structure suitable for subsequent experiments.

### 3.8 MeV can effectively reduce the level of inflammatory factors in Caco-2 cells

The ELISA results (**Figure 7D**) showed that Erastin increased pro-inflammatory cytokines TNF-α, IL-6, and IL-8 compared to the CON group (P<0.001). Both the MeV and Ferrostatin-1 groups reversed these effects (all P<0.05 vs Erastin group). However, both the low- and high-dose MeV groups showed statistically significant differences in therapeutic efficacy when compared to the Fer-1 group.

### 3.9 MeV can repair the mucosal barrier function of Caco-2 cells

Western blotting analyses demonstrated that Erastin downregulated Occludin and ZO-1 expression compared to the controls (P<0.001). Both MeV and Fer-1 treatment effectively restored their expression (P<0.001). However, there were also statistically significant differences between the MeV and Fer-1 groups (P<0.001) (**Figure 7E**).

RT-qPCR results revealed that compared to the CON group, the Erastin group exhibited significantly reduced mRNA expression levels of Occludin and ZO-1 (P<0.001). Intervention with both MeV and Fer-1 reversed this change to varying degrees. The disparity between the low-dose MeV group and the Erastin group was not significantly different (P>0.05), both the high-dose MeV group and the Fer-1 group showed highly significant differences compared to the Erastin group (P<0.001) (**Figure 7F**).

### 3.10 MeV can effectively regulate the level of oxidative stress in Caco-2 cells

**Figure 8:**
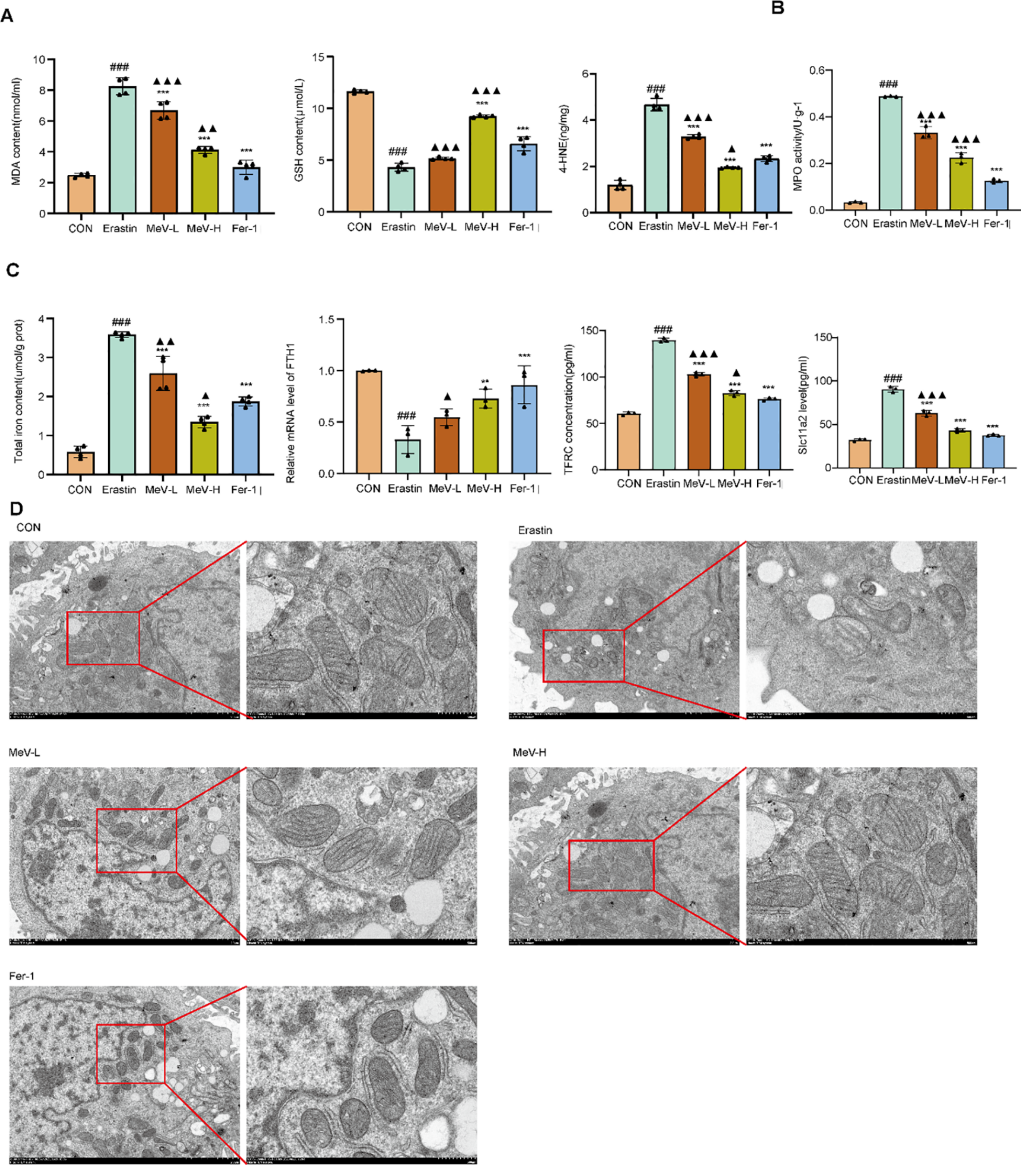
MeV stabilizes oxidative stress levels, Iron metabolic state and mitochondrial status in Caco-2 cells. **(A)** MDA,GSH and 4-HNE content. **(B)** MPO activity. **(C)** Iron metabolic state **(D)** Mitochondrial morphology observed by TEM (×7.0K; × 20.0K). Data are presented as mean ± SD. ###p < 0.001 versus CON group; **p < 0.01, and ***p < 0.001 versus Erastin group, ▴P<0.05, ▴▴p < 0.01, ▴▴▴p < 0.001 versus Fer-1 group.

Erastin treatment increased the levels of MDA and 4HNE level (P<0.001) and decreased GSH level compared to the CON group (P<0.001). Both MeV and Ferrostatin-1 effectively reversed these changes. Notably, the high-dose MeV group demonstrated superior efficacy compared to the MeV-L group (**Figure 8A**). The MPO activity in the Erastin group was significantly higher than that in the CON group, while interventions with both MeV and Fer-1 reduced MPO expression, thereby reversing the abnormal elevation trend (**Figure 8B**).

### 3.11 MeV effectively inhibits ferroptosis in Caco-2 cells

Fe content was significantly higher in the Erastin group than in the CON group (P < 0.001). MeV and Fer-1 groups exhibited a decrease in Fe content compared with the Erastin group (P < 0.001). And the MeV-H group demonstrated superior efficacy compared to the MeV-L group. Additionally, we measured the levels of FTH1, TFRC, and Slc11a2 in cells. Compared with the CON group, the FTH1 level was significantly decreased in the Erastin group, while the levels of TFRC and Slc11a2 were significantly increased. Interventions with MeV and Fer-1 reversed the trends observed in the Erastin group to varying degrees. After intervention, the expression patterns shifted closer to those of the CON group, thereby restoring iron metabolic homeostasis (**Figure 8C**).

In the control group, the cells exhibited abundant mitochondria with normal size and morphology, clearly defined double-membrane structures, and densely packed, regularly arranged cristae. In contrast, the Erastin group showed enlarged and severely swollen mitochondria with distorted morphology, extensive dissolution of the double-membrane structure, blurred boundaries, and only a few disorganized cristae. Although the low-dose MeV group exhibited some recovery in mitochondrial damage compared to the Erastin group, the overall damage remained substantial. The high-dose MeV group displayed mitochondria of normal size, with minor dissolution of the membrane and a relatively high number of cristae, albeit mildly dilated. And the Fer-1 group demonstrated mitochondria of normal size, clear double-membrane boundaries, and an increased number of cristae, although their arrangement remained slightly disordered (**Figure 8D**).

**Figure 9:**
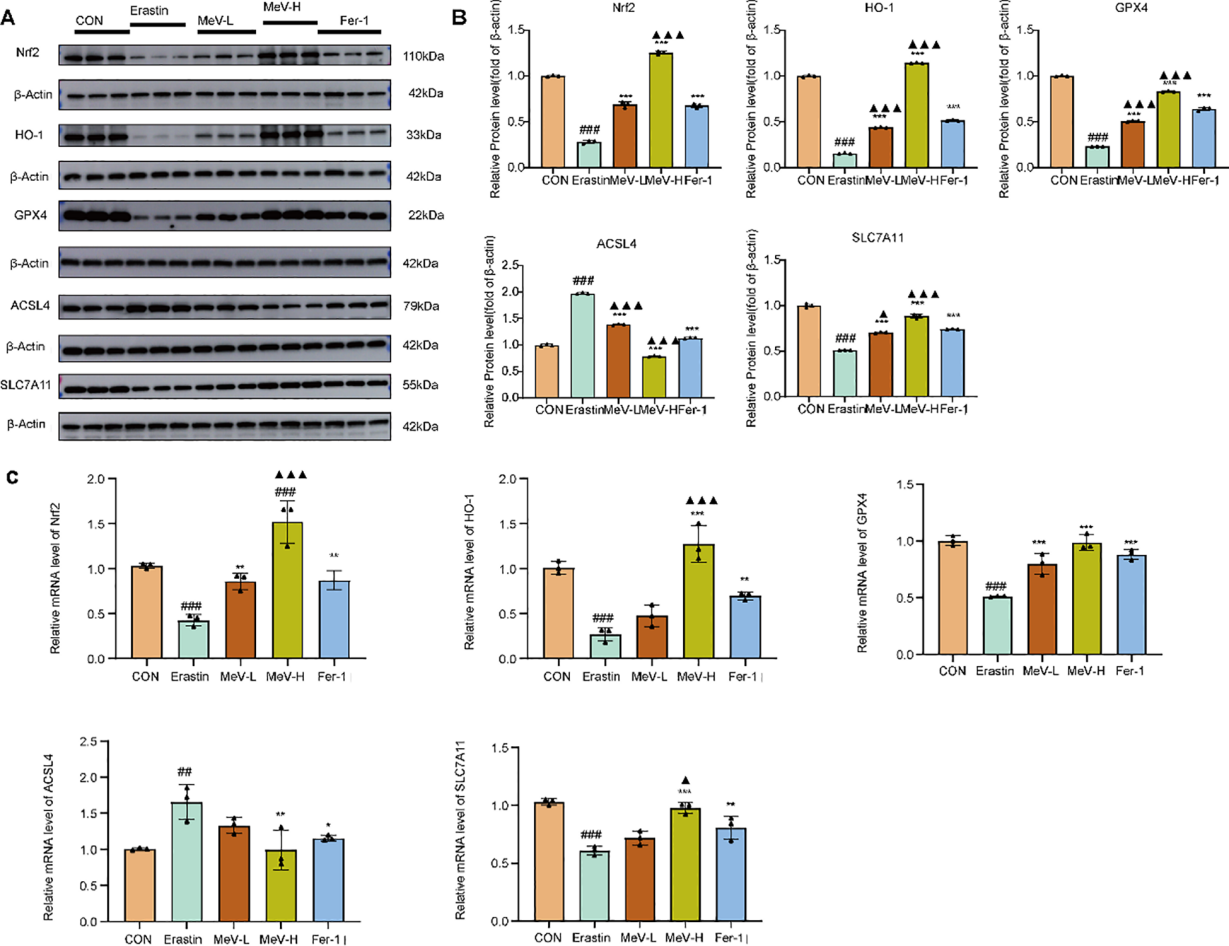
MeV modulates ferroptosis and the Nrf2/HO-1 signaling pathway in Caco-2 cells. **(A, B)** Western blot and quantification. **(C)** Relative mRNA levels of indicators. Data are presented as mean ± SD. #P<0.05, ## p < 0.01, ###p < 0.001 versus CON group; *p < 0.05, **p < 0.01, and ***p < 0.001 versus Erastin group; ▴P<0.05, ▴▴p < 0.01, ▴▴▴p < 0.001 versus positive control(Fer-1)group.

Western blotting results revealed that compared to the CON group, the Erastin group exhibited significantly decreased expression of GPX4 and SLC7A11, while ACSL4 expression was markedly upregulated (P < 0.001). Compared to the Erastin group, both MeV-L and MeV-H groups and the Fer-1 group showed substantial recovery in GPX4 and SLC7A11 expression, along with a substantial decrease in ACSL4 levels (P < 0.001) (**Figures 9A, B**).

RT-qPCR results revealed that compared to the CON group, the Erastin group exhibited significantly decreased level of GPX4 and SLC7A11, along with markedly upregulated ACSL4 expression (P < 0.001, P < 0.001, P < 0.01). In comparison to the Erastin group, both MeV and Fer-1 groups showed increased expression of GPX4 and SLC7A11, with the MeV-H group demonstrating a more substantial recovery than the MeV-L group. Although ACSL4 expression was reduced in both MeV and Fer-1 groups relative to the Erastin group, the difference observed in the low-dose MeV group was not statistically significant (P>0.05). In contrast, the MeV-H and Fer-1 groups exhibited highly significant differences in ACSL4 expression compared to the Erastin group (P<0.01, P<0.05) (**Figure 9C**).

### 3.12 MeV can regulate the Nrf2/HO-1 signaling pathway in Caco-2 cells

Western blot examination demonstrated that, compared to the CON group, the Erastin group displayed a markedly decreased expression of Nrf2 and HO-1. (P < 0.001). Relative to the Erastin group, the low-dose MeV group, high-dose MeV group, and Fer-1 group all demonstrated markedly increased expression of Nrf2 and HO-1 (P < 0.001 for all), with the MeV-H group showing the most pronounced upregulation (**Figures 9A, B**).

RT-qPCR results revealed that compared to the CON group, the Erastin group exhibited significantly decreased expression of Nrf2 and HO-1 (P < 0.001). Relative to the Erastin group, both the MeV and Fer-1 groups showed elevated expression of Nrf2 and HO-1 to varying degrees, with the MeV-H group demonstrating the most pronounced upregulation (P < 0.001 for both) (**Figure 9C**).

### 3.13 Regulation of ferroptosis in Caco-2 cells by MeV is critically dependent on the Nrf2/HO-1 pathway

**Figure 10:**
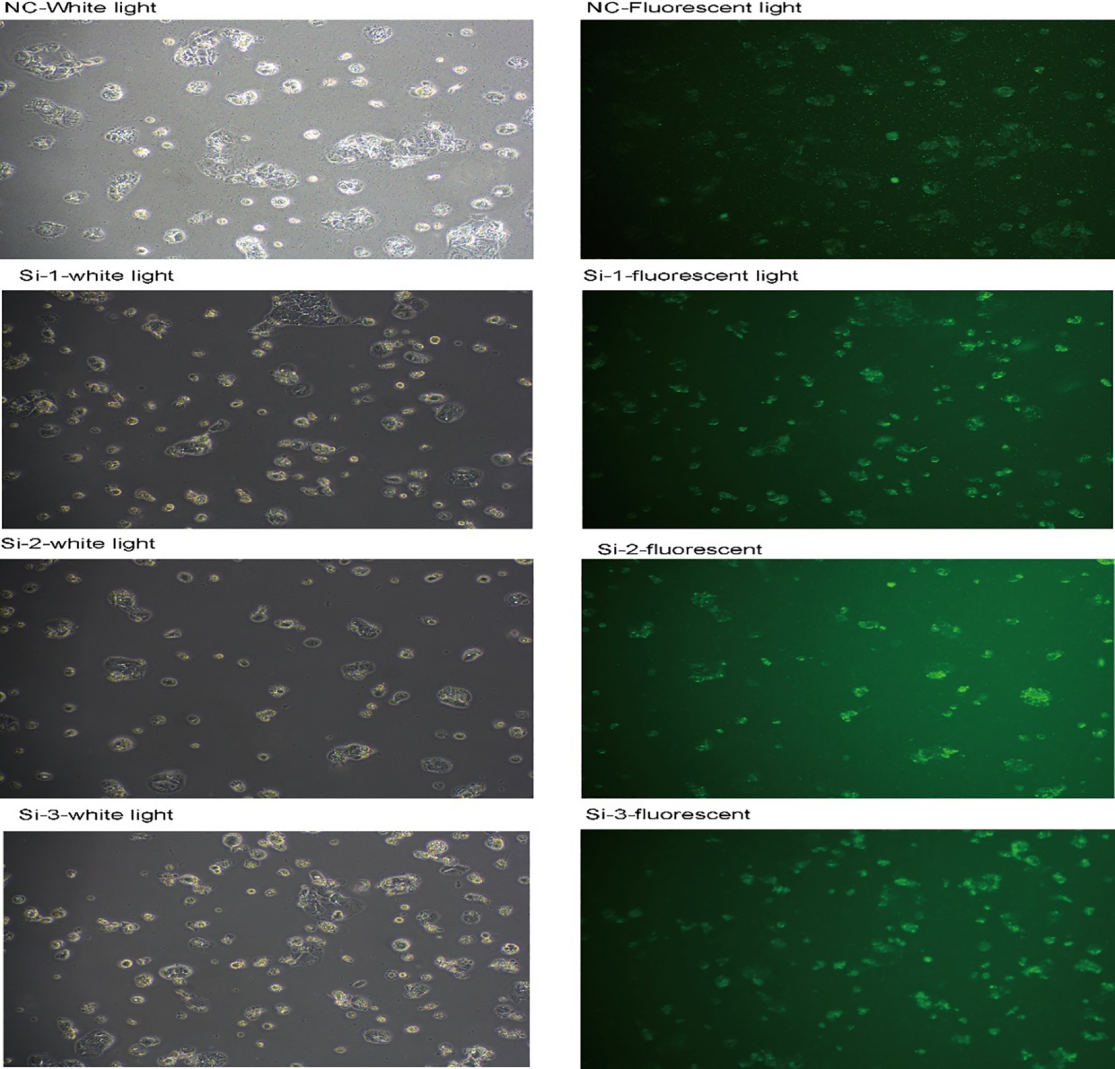
Transfection status was observed under a fluorescence microscope.

**Figure 11:**
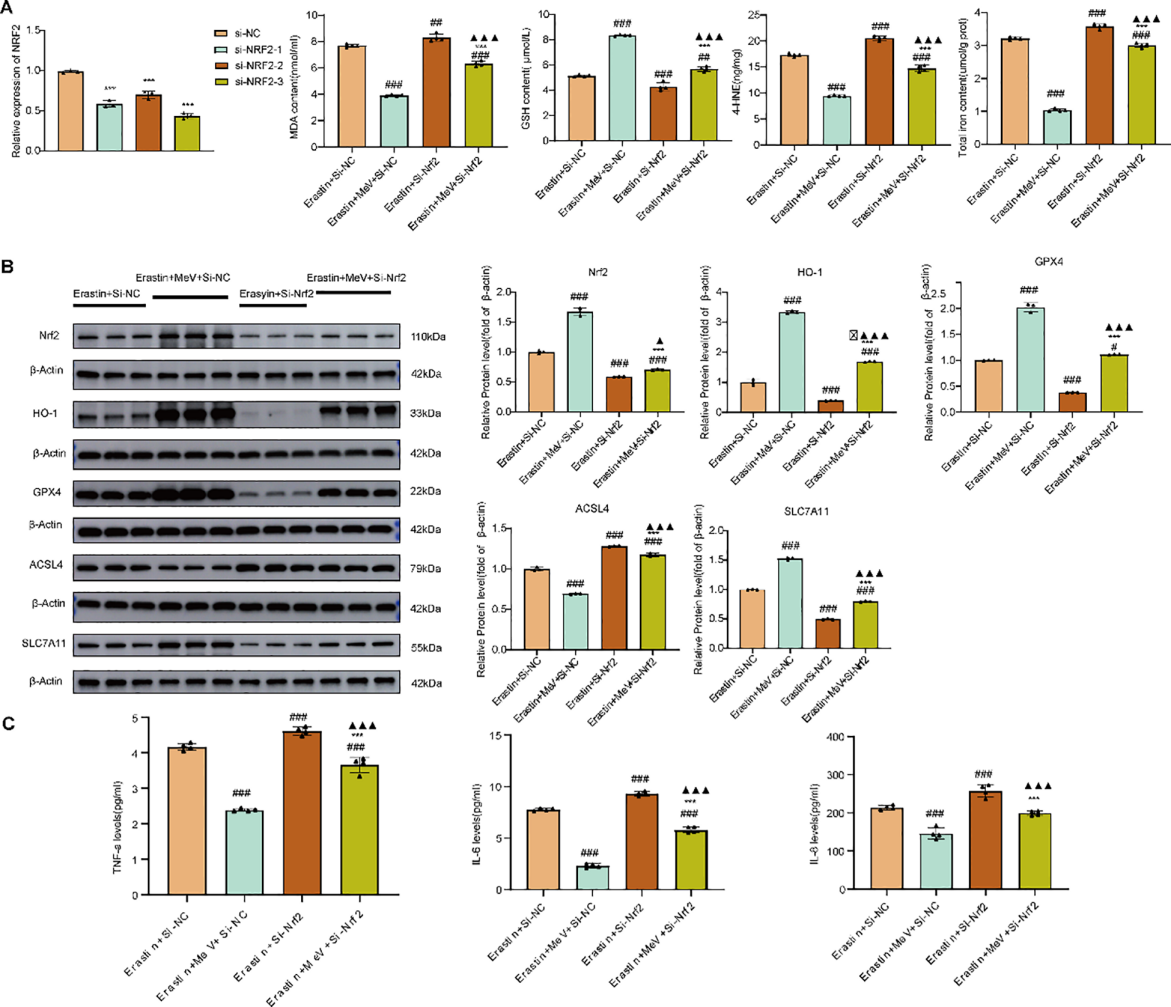
Changes in downstream signaling markers and inflammatory cytokine levels following successful Nrf2 knockdown in Caco-2 cells. **(A)** Comparative analysis of Nrf2 mRNA expression levels among three transfection sequences versus NC group; Oxidative stress parameters and Fe content. **(B)** Western blot and quantification. **(C)** Inflammatory factor expression levels. Data are presented as mean ± SD. #P<0.05, ## p < 0.01, ###p < 0.001 versus Erastin + Si-NC group; *p < 0.05, **p < 0.01, and***p < 0.001 versus Erastin + MeV +Si-NC group; ▴p < 0.05, ▴▴▴p < 0.001 versus Erastin+Si-Nrf2 group.

Screening for optimal transfection sequences: Following transfection with three distinct Nrf2 siRNA sequences, the transfection process was observed using a fluorescence microscope (**Figure 10**). RT-qPCR analysis demonstrated significantly reduced Nrf2 mRNA expression across all siRNA groups compared to that in the Si-NC group (P < 0.001). Among these, siRNA-3 exhibited the most potent Nrf2 suppression compared with siRNA-1 and siRNA-2 (**Figure 11A**). Based on these results, siRNA-3 was selected for lipid-encapsulated oligonucleotide transfection in subsequent experiments.

Following successful Nrf2 knockdown, oxidative stress parameters were measured in each group. The results demonstrated that MeV intervention significantly reduced the expression levels of MDA, 4-HNE, and Fe compared to the Erastin + Si-NC group (P < 0.001 for all). In contrast, the Erastin+Si-Nrf2 group showed significantly increased expression of MDA, 4-HNE, and Fe relative to the Erastin + Si-NC group (P<0.01, P < 0.001, P < 0.001). Although the expression of these three indicators in the Erastin+MeV+Si-Nrf2 group was decreased compared to both the Erastin + Si-NC and Erastin+Si-Nrf2 groups, it remained significantly higher than that in the Erastin+ MeV + Si-NC group (P<0.001 for all). Compared to the Erastin +Si-NC group, the Erastin + MeV + Si-NC group demonstrated significantly elevated GSH expression (P < 0.001). In contrast, the Erastin+Si-Nrf2 group exhibited lower GSH levels than the Erastin +Si-NC group (P < 0.001). Although the Erastin+MeV+Si-Nrf2 group showed higher GSH expression compared to both the Erastin + Si-NC (P < 0.01) and Erastin+Si-Nrf2 (P < 0.001) groups, it remained somewhat inferior to that in the Erastin + MeV + Si-NC group (P < 0.001) (**Figure 11A**).

Western blot results revealed that compared to the Erastin + Si-NC group, the Erastin + MeV + Si-NC group exhibited significantly elevated protein expression of Nrf2, HO-1, GPX4 and SLC7A11 (P<0.001). In contrast, the Erastin+Si-Nrf2 group showed markedly decreased expression of these proteins (P< 0.001). Although the expression levels in the Erastin+MeV+Si-Nrf2 group were higher than those in the Erastin+Si-Nrf2 group, they remained significantly lower compared to the Erastin + MeV + Si-NC group (P< 0.001 for all). Compared to the Erastin + Si-NC group, the Erastin + MeV+ Si-NC group exhibited significantly decreased ACSL4 expression (P<0.001), while the Erastin+Si-Nrf2 group showed markedly increased ACSL4 levels (P<0.001). Although ACSL4 expression in the Erastin+MeV+Si-Nrf2 group was decreased compared to the Erastin+Si-Nrf2 group (P<0.001), it remained markedly elevated compared to the Erastin + MeV +Si-NC group (P<0.001) (**Figure 11B**).

ELISA results revealed that compared to the Erastin + Si-NC group, the Erastin + MeV + Si-NC group demonstrated markedly reduced expression of TNF-α, IL-6, and IL-8 (P<0.001 for all). In contrast, the Erastin+Si-Nrf2 group demonstrated markedly elevated expression of these three cytokines (P<0.001 for all). Although the levels of TNF-α, IL-6, and IL-8 in the Erastin+MeV+Si-Nrf2 group were reduced compared to the Erastin+Si-Nrf2 group (P<0.001 for all), they remained substantially greater than those in the Erastin + MeV + Si-NC group (P<0.001 for all) (**Figure 11C**). Indicating that MeV effects are partially attenuated by knockdown.

## 4 Discussion

In recent years, herbal medicines and their isolated chemical constituents have garnered growing recognition from both researchers and patients, as well as endorsement from the WHO. This is due to their superior safety profile, lower cost, and promising efficacy in the treatment of diseases such as inflammatory bowel disease, particularly when compared to conventional pharmaceutical options (22, 23). Current research on the anti-inflammatory mechanisms of MeV has primarily focused on the TNF/MAPK/NF-κB and RAGE/MEK/ERK pathways, the relationship between MeV and ferroptosis, as well as the Nrf2/HO-1 pathway, has not yet been explored. This study bridges this gap by using a UC model with MeV intervention, combining *in vivo* and *in vitro* validations. Knockdown of Nrf2 using siRNA confirmed the critical role of this pathway. These results demonstrate MeV’s dual mechanism: regulation of ferroptosis via Nrf2/HO-1, promotes intestinal mucosal barrier repair thus providing novel therapeutic evidence for UC.

UC animal models can be induced by various methods including DSS, trinitrobenzene sulfonic acid, and acetic acid, with the DSS-induced model is the greatest widely utilized and best-established approach (24). It closely mimics human UC manifestations and colonic damage (25). DAI scoring, spleen index, H&E staining and colon length remain gold standards for evaluating UC severity and therapeutic efficacy (26, 27). In this study, DSS-treated rats showed characteristic UC manifestations: elevated DAI scores, spleen index increases, colon shortening, and histopathological damage with elevated inflammation, confirming successful model establishment (28–30). After MeV intervention, the above pathological symptoms of rats were significantly improved and tended to be normal. Demonstrating that MeV effectively alleviated inflammatory responses in UC rats.

The intestinal mucosal barrier act as the host-environment interface, protects against harmful substances, and maintains homeostasis (31, 32). Goblet cells secrete MUC2 mucin to form the mucus barrier (33). DSS treatment substantially reduced goblet cells and disrupted this layer, whereas MeV restored mucosal structure and increased goblet cell numbers. Tight junction proteins, such as Occludin and ZO-1, whose reduction increases UC risk, were significantly upregulated by MeV. These results suggest that MeV facilitates the restoration of intestinal mucosal barrier function and ameliorates inflammatory damage.

Ferroptosis is an iron-dependent, non-apoptotic type of regulated cell death that is distinct from conventional apoptosis and autophagy, has been increasingly implicated in modulating the inflammatory activity in UC (34, 35). Intestinal epithelial cells, integral to the intestinal mucosal barrier, are crucial for maintaining normal gut function. Ferroptosis in these cells compromises intestinal barrier function, precipitates a cascade of abnormal immune responses, and contributes to the development of colitis (36, 37). Accumulating research has established the involvement of ferroptosis in the onset and progression of inflammatory and autoimmune diseases (38, 39). Convincing evidence of ferroptosis has been identified in colonic tissue samples from UC patients and in UC animal models (40, 41). Thus, targeted inhibition of intestinal epithelial cell death to restore epithelial barrier integrity represents a significant therapeutic strategy for colitis. A bidirectional regulatory relationship exists between ferroptosis and oxidative stress (OS). Collapse of the antioxidant system and accumulation of lipid peroxides trigger ferroptosis, which in turn releases pro-oxidant substances, propagates oxidative damage, and establishes a vicious cycle that amplifies OS (42). Therefore, targeting the OS–ferroptosis axis is crucial for UC prevention and treatment. GPX4, a key glutathione peroxidase, reduces phospholipid hydroperoxides to prevent ferroptosis; its inactivation impairs antioxidant defense (43). Studies have demonstrated that ACSL4 serves as a key biomarker and driver of ferroptosis, which counteracts cell death through remodeling lipid composition. In resisting lipid peroxidation, ACSL4-knockout cells exhibit comparable resistance to ferroptosis. Unlike GPX4, ACSL4 positively regulates ferroptosis (44). SLC7A11 mediates cystine uptake for GSH synthesis, with deficiency promoting lipid peroxide accumulation (45). These proteins directly indicate ferroptosis status. In our study, DSS or Erastin group decreased expression of these markers, elevated Fe, and mitochondrial damage versus control group, but all reversed by MeV treatment, demonstrating effective ferroptosis suppression.Nrf2, a pivotal antioxidant transcription factor, modulates three key ferroptosis pathways: Synthesis and metabolism of GSH/GPX4, iron absorption, and lipid peroxidation by transcriptionally regulating multiple ferroptosis-related genes (46). Upon attenuation of Nrf2 ubiquitination and degradation, nuclear Nrf2 accumulation activates the transcription of downstream antioxidant enzymes—including HO-1 and GPX4—through their interaction with antioxidant response components, thereby maintaining systemic redox homeostasis (47). HO-1 exhibits dual anti-inflammatory effects by suppressing cytokines and producing anti-inflammatory carbon monoxide during heme catabolism (48). In this study, model group exhibited significantly reduced Nrf2 and HO-1 expression, whereas MeV intervention upregulated them, even surpassing levels in the control group. These results demonstrate that MeV activates the Nrf2/HO-1 signaling pathway, alleviates oxidative stress pathology, thus suppresses ferroptosis progression.

The specific mechanisms underlying MeV’s effects on UC were explored using multidimensional *in vivo* and *in vitro* experiments. The research features comprehensive content, substantial workload, and robust data, all of which strongly support the conclusions.

## 5 Limitations

This study has several limitations. First, the findings are based on an acute experimental colitis model in male Sprague–Dawley rats; disease chronicity, relapse–remission dynamics, and potential sex differences were not assessed. Second, although we provide pathway evidence consistent with Nrf2/HO-1–linked ferroptosis restraint, causal inference *in vivo* remains incomplete because genetic or pharmacologic gain/loss-of-function of ferroptosis (e.g., GPX4 modulation, *in vivo* ferroptosis inhibitors) was not performed. Third, mechanistic validation in epithelial organoids and immune-epithelial co-culture systems was not included, limiting insights into cell–cell crosstalk. Fourth, we did not conduct pharmacokinetic/pharmacodynamic, metabolism, or toxicology evaluations, so exposure–response relationships and safety margins for 5-O-methylvisammioside (MeV) remain to be defined. Fifth, *in vitro* assays relied primarily on Caco-2 cells, which may not fully recapitulate primary epithelial biology; confirmation in primary tissues or patient biopsies is warranted. Finally, we did not analyze microbiome composition or mucosal immunophenotypes, which could interact with ferroptosis and redox signaling. Peripheral/systemic inflammatory markers may not fully represent intramucosal immune microenvironment changes, we will position immune-cell profiling and microbiome analyses as priority next steps.Therefore, these gaps should be addressed in chronic models including both sexes, with rigorous causal and translational studies.

## 6 Conclusions

In summary, 5-O-methylvisammioside (MeV) attenuated experimental colitis, improving clinical indices and histopathology while preserving epithelial barrier markers. Across *in vivo* and cell-based assays, MeV activated the Nrf2/HO-1 axis, limited lipid peroxidation and iron overload, and restrained ferroptosis-associated changes in antioxidant defenses (e.g., GPX4/ACSL4), consistent with partial restoration of redox homeostasis. Although the present data support an Nrf2/HO-1–linked mechanism, definitive *in vivo* causality requires targeted gain/loss studies. Together, these results identify MeV as a ferroptosis-targeting, antioxidant candidate for colitis and provide a mechanistic framework to guide dose selection, PK/PD, safety profiling, and validation in chronic, sex-balanced models and human-relevant systems.

## Data Availability

The raw data supporting the conclusions of this article will be made available by the authors, without undue reservation.
